# Drug effectiveness for COVID-19 inpatients inferred from Japanese medical claim data using propensity score matching

**DOI:** 10.12688/f1000research.131102.1

**Published:** 2023-04-13

**Authors:** Shingo Mitsushima, Hiromasa Horiguchi, Kiyosu Taniguchi

**Affiliations:** 1 Center for Field Epidemic Intelligence, Research and Professional Development, National Institute of Infectious Diseases, Shinjuku-ku, Tokyo, 1620052, Japan; 2 Department of Clinical Data Management and Research, Clinical Research Center, National Hospital Organization, Meguro-ku, Tokyo, 1528621, Japan; 3 General Director, National Hospital Organization Mie National Hospital, Tsu, Mie, 5140125, Japan; 4 Research Director, The Tokyo Foundation for Policy Research, Minato-ku, Tokyo, 1066234, Japan

**Keywords:** COVID-19, mutated strain, underlying diseases, antibody cocktail, antiviral drug, mortality, steroid and anti-inflammatory drug

## Abstract

**Background:** Earlier studies and clinical trials have shown that the drugs such as antiviral drugs, antibody cocktails, and steroids and anti-inflammatory drugs are expected to prevent severe coronavirus 2019 (COVID-19) outcomes and death.

**Methods:** We used observational data for Japan to assess the effectiveness of these drugs for COVID-19. We applied propensity scoring, which can treat the choice of administered drug as a random assignment to inpatients, to the Medical Information Analysis Databank operated by National Hospital Organization in Japan. The outcome was defined as mortality. Subjects were all inpatients, inpatients with oxygen administration, and inpatients using respiratory ventilators, classified by three age classes: all ages, 65 years old or older, and younger than 65 years old. Information about demographical characteristics, underlying disease, administered drug, the proportion of Alpha, Beta and Omicron variant strains, and vaccine coverage were used as explanatory variable in logistic regression.

**Results:** Estimated results indicated that only an antibody cocktail (sotrovimab, casirivimab and imdevimab) raised the probability to save life consistently. By contrast, other drugs might reduce the probability of saving life. The results indicated that an antiviral drug (remdesivir), a steroid (dexamethasone), and an anti-inflammatory drug (baricitinib and tocilizumab) might not contribute to saving life even at the pseudo-situation of random assignment. However, this logistic regression at the first step might have only insufficient explanatory power.

**Conclusions:** We found a high likelihood that antibody cocktails were consistently effective to raise the probability of saving life, though a lesser likelihood in other drugs for older patients with mild to severe severity and all age patients with moderate severity.

## Introduction

Coronavirus disease 2019 (COVID-19) is caused by severe acute respiratory syndrome coronavirus 2 (SARS-CoV-2). COVID-19 caused pandemic throughout the world from December 2019. In Japan, the first case of COVID-19 was detected in January 2020, and then COVID-19 spread throughout Japan. As characteristics of COVID-19 were revealed and treatments for COVID-19 developed, the mortality rate due to COVID-19 in Japan has declined. ^1^

Earlier studies and clinical trials have shown that drugs used against COVID-19, such as antiviral drugs (remdesivir), ^2^ antibody cocktails (casirivimab/imdevimab and sotrovimab), ^3^^,^^4^ steroids (dexamethasone), ^5^ and anti-inflammatory drugs (baricitinib and tocilizumab), ^6^^–^^8^ statins, ^9^^,^^10^ RNA-dependent RNA polymerase inhibitor (molnupiravir), ^11^^,^^12^ protease inhibitor (nirmatrelvir/ritonavir), ^11^^,^^13^ are expected to prevent severe COVID-19 outcomes and death. For this study, we considered the real-world effectiveness of drugs against COVID-19.

The National Hospital Organization (NHO) in Japan, an organization of regional core hospitals accounting for about 3.4% of all beds in Japan, ^14^ provides a database of medical claims from 60 representative NHO hospitals: the Medical Information Analysis Databank (MIA). NHO is one of the largest organizations in Japan and each prefecture has one or more NHO hospitals. MIA collects the data on medical insurance claims for outpatients and inpatients. It includes inpatients’ demographical characteristics, underlying diseases, medical interventions including oxygen administration, the use of respiratory ventilators, administration of drugs, and outcomes such as discharge or death. ^15^ We used MIA data of inpatients to verify drug effectiveness. MIA does not have the data on vaccination history or causative strain.

In the real world, whether a drug is administered or not depends on the patient condition. In other words, administration of drugs is probably not a random assignment. Generally speaking, patients who have a higher likelihood of developing an illness have a higher probability of being administered drugs, which implies, because of selection bias in the observational data, a negative association between drug administration and outcomes. Therefore, non-random choice of drug administration should be taken into account to estimate the drug effectiveness. Propensity score matching might resolve this problem statistically but not experimentally. ^16^ This procedure predicts the likelihood of receiving drugs initially. Then outcomes are compared between a drug-received group and a drug not-administered group, the members of which have an almost identical likelihood of having been administered some drugs. We then examined drug effectiveness using this method.

Propensity score matching is widely used in social sciences for the evaluation of programs: participants choose to join a program spontaneously, such as a job training program or an unemployment payment program. However, in the natural sciences, a researcher can perform experiments and thereafter delete selection bias in the choice of subjects. Nevertheless, such experiments are expensive and require a longer period. Moreover, the number of subjects should be constrained; outcomes tend to be evaluated under less severe condition. ^17^^,^^18^

In medicine, random assignment experiments after launching are not so popular. Therefore, situations change, mutated strains emerge, vaccine coverage and/or developing treatment might affect drug effectiveness, making it impossible to evaluate. In particular, mortality tends not to be used as an outcome for evaluation, even though it should be the endpoint of greatest concern. Therefore, experiments in medicine for updated situations and mortality might be difficult. In other words, propensity score matching methods might be useful even for medicine, particularly for orthopaedic surgery and cardiovascular research. ^17^^,^^18^

## Method

### Data sources

This study used MIA for confirmed inpatients including their age, sex, underlying diseases, hospitalization date, administered drug, outcome and whether they received oxygen therapy and/or ventilation.

We used data for vaccine administration published by the Cabinet Secretariat. Moreover, prevalence in Alpha, Delta and Omicron variant strains were referred from a monitoring meeting in Tokyo because MIA included no information about the patient’s vaccine status and sublineage in SARS-CoV-2.

The study period was January 2020 through March 2022, using data recorded as of May 2022. This study area was the entirety of Japan.

### Definitions of variables

We defined demographical conditions as age and sex, underlying diseases as cancer (C00–C90 in ICD10), asthma (J45), chronic obstructive pulmonary disease (COPD) (J44), hypertension (HT) (I10), heart failure (HF) (I50), and diabetes mellitus (DM) (E10). We examined the effects of an antiviral drug (remdesivir), ^2^ an antibody cocktail (sotrovimab and casirivimab/imdevimab), ^3^^,^^4^ a steroid (dexamethasone), ^5^ and anti-inflammatory drugs (baricitinib and tocilizumab), ^6^^–^^8^ which were proven effective against COVID-19 by previous studies. Because the number of cases with antibody cocktails was insufficiently large, we did not divide them by the name of drug, as sotrovimab, casirivimab, and imdevimab. We considered the overall effects of antibody cocktails. These drugs were the five most common drugs against COVID-19. We assumed that vaccination coverage was the rate of the second dose of vaccine received two weeks prior by age class, as younger than 65 years old, and 65 years old or older. Mutated strains, Alpha, Delta and Omicron variant strains, were measured by percentage at one week before admission. The Alpha, Delta and Omicron variant strains were defined as the proportion of Alpha, Delta and Omicron variant strains. Omicron included BA.2 or a later sublineage. Alternatively, we used a dummy variable for the period of the 4–6th wave instead of the proportion of the mutated strains as an explanatory variable, to check robustness. By this specification, the Alpha variant strain emerged and then dominated in the 4th wave, defined as from 1 March through 20 June 2021. Similarly, the Delta variant strain emerged and then dominated in the 5th wave, defined as 21 June through 21 November 2021. Omicron BA.1 strain emerged and then dominated from the 6th wave, defined as 22 November 2021 to the end of the study period. Death during hospitalization was defined as the outcome.

### Subjects

Subjects were all inpatients confirmed as having SARS-CoV-2. However, aside from pure medical criteria, the criteria of hospitalization for asymptomatic patients or patients with mild symptoms who did not require oxygen therapy were probably affected strongly by medical resource scarcity or social situations such as support for their staying at home and recuperation at home. For that reason, we also limited our study to subjects who were expected to be inpatients with oxygen therapy or respiratory ventilation.

### Statistical analysis

We conducted estimates separately by the type of drug: remdesivir, antibody cocktail (sotrovimab, casirivimab and imdevimab), dexamethasone, baricitinib, and tocilizumab.

The first step to assess whether the patient was administered a type of drug or not was performed through logistic regression on their age, sex, underlying diseases, pharmaceutical therapy, vaccine coverage, and prevalence in the mutated strains as explanatory variables. The second step was comparison of outcomes among administered patients and unadministered patients with almost identical likelihood as the prediction done in the first step.

All statistical analyses were conducted using Stata software (SE 17.0; Stata Corp.). We adopted 5% as the significance level.

### Ethical considerations

Individual informed consent was not required to conduct this study because the dataset was provided as anonymized data by National Hospital Organization. ^15^ This study was approved by the Ethics Committee of Mie Hospital (Approval No. 2020-89) on 23 October 2020. Specific permission to use MIA data was obtained from the NHO (Registration No. 1201003) on 1 December 2020.

## Results

A supplementary table (see *Extended data* ^19^), presents a summary of the estimation results obtained for the first step logistic regression for drug administration. Table 1 presents the estimation results obtained for all inpatients, inpatients with oxygen therapy, and inpatients who used a respiratory ventilator in three age classes. Results show that 24 significance estimators of 90 estimators in all, but 20 estimators were positive, which indicates that drug administration reduced the probability of saving life.

**Table 1.** Estimation results of propensity score matching method.

Age class	All		65 years old and older		Younger than 65 years old	
	difference	*p*-value	difference	*p*-value	difference	*p*-value
Remdesivir						
Proportion						
All	0.034	0.000	0.064	0.000	0.019	0.068
Oxygen administration	0.015	0.315	0.045	0.056	0.013	0.311
Respiratory ventilator used	0.012	0.768	0.127	0.080	N.A.	N.A.
Period						
All	0.031	0.000	0.067	0.004	0.010	0.126
Oxygen administration	0.012	0.348	0.044	0.087	0.011	0.337
Respiratory ventilator used	-0.006	0.893	0.047	0.477	N.A.	N.A.
Dexamethasone						
Proportion						
All	0.027	0.000	0.079	0.000	0.003	0.253
Oxygen administration	0.020	0.013	0.052	0.000	0.006	0.234
Respiratory ventilator used	-0.021	0.548	-0.055	0.296	0.044	0.319
Period						
All	0.029	0.000	0.076	0.000	0.004	0.119
Oxygen administration	0.014	0.090	0.038	0.011	0.006	0.131
Vent	-0.051	0.158	-0.083	0.125	0.008	0.857
Tocilizumab						
Proportion						
All	0.091	0.000	0.214	0.000	0.019	0.057
Oxygen administration	0.079	0.001	0.126	0.021	N.A.	N.A.
Respiratory ventilator used	0.117	0.227	N.A.	N.A.	-0.077	0.030
Period						
All	0.103	0.000	0.223	0.000	0.025	0.030
Oxygen administration	0.086	0.001	0.126	0.020	N.A.	N.A.
Respiratory ventilator used	0.090	0.375	N.A.	N.A.	-0.062	0.079
Baricitinib						
Proportion						
All	0.023	0.100	0.411	0.114	N.A.	N.A.
Oxygen administration	-0.016	0.220	-0.033	0.900	-0.008	0.023
Respiratory ventilator used	-0.149	0.586	0.384	0.000	N.A.	N.A.
Period						
All	N.A.	N.A.	N.A.	N.A.	N.A.	N.A.
Oxygen administration	N.A.	N.A.	N.A.	N.A.	N.A.	N.A.
Respiratory ventilator used	N.A.	N.A.	N.A.	N.A.	N.A.	N.A.
Sotrovimab or casirivimab/imdevimab						
Proportion						
All	N.A.	N.A.	-0.105	0.000	N.A.	N.A.
Oxygen administration	-0.085	0.000	N.A.	N.A.	N.A.	N.A.
Respiratory ventilator used	N.A.	N.A.	N.A.	N.A.	N.A.	N.A.
Period	N.A.	N.A.	N.A.	N.A.	N.A.	N.A.
All	N.A.	N.A.	N.A.	N.A.	N.A.	N.A.
Oxygen administration	N.A.	N.A.	N.A.	N.A.	N.A.	N.A.
Respiratory ventilator used	N.A.	N.A.	N.A.	N.A.	N.A.	N.A.

**Abbreviations:** N.A., not available.

**Notes**: Yellow denotes significance.

Four estimators indicating that a drug probably contributes to saving life were the following: tocilizumab among younger inpatients with ventilators; baricitinib among younger inpatients with oxygen therapy; and antibody cocktails among all older inpatients and among all inpatients with oxygen therapy. Of three drugs, only the antibody cocktail did not have a positive difference, which suggests an increase in the probability of saving life. No other treatment effect estimator of such drugs was available. Some treatment effects in tocilizumab and baricitinib were positive, especially for all older inpatients or older inpatients with moderate severity. Therefore, the results obtained for these two drugs were mixed.

## Discussion

Results showed that antibody cocktails might contribute to saving life. The estimated results for tocilizumab and baricitinib were mixed, but these drugs were presumed to have some probability of saving life among younger but severe patients. However, we were unable to find any evidence that remdesivir and dexamethasone contribute to saving life at all.

These counterintuitive findings, which are inconsistent with results of earlier studies of the effectiveness of remdesivir and dexamethasone, might result from worse matching at the first step. ^2^^,^^5^ Also, endogeneity might not be controlled well in decisions to use drugs. Drugs to be administered against COVID-19 depend on the severity of illness of the patients. For instance, an earlier study showed dexamethasone as effective for moderate or severe COVID-19, but ineffective for mild COVID-19. ^5^ Actually, more than antibody cocktails, ^3^^,^^4^ remdesivir ^2^ and dexamethasone ^5^ tend to be administered more for severer patients. To resolve this difficulty, more information indicating severity is needed. Moreover, we did not use test results such as blood pressure, body mass index, and oxygen saturation. We must include such information in the first step. Inclusion of such data remains a challenge for future research.

The results for antibody cocktails included many “not available” entries, although drug effectiveness was confirmed in well matched cases. That finding might be attributable to the small sample of antibody cocktail use for patients. In Japan, remdesivir, baricitinib, casirivimab/imdevimab, sotrovimab, and tocilizumab were approved for treatment of COVID-19, respectively, in May 2020, April 2021, July 2021, September 2021, and January 2022. ^20^ More recently approved drugs tend to be associated with smaller samples. Data accumulation might resolve that shortcoming.

### Limitations

First, we estimated the effectiveness of drugs separately. However, the choice of drug was not actually independent. Therefore, intercorrelation among drugs should be incorporated into the estimation model.

Second, because MIA is a database of medical claims, data of the prior few weeks might change during a few months. For this study, we collected and analyzed data from January 2020 through the end of March 2022, recorded as of May 2022. Data and therefore estimation results might change over time if the study period were extended. In addition to this, we examined using Japanese vaccine coverage instead of the vaccination history of the patients. We also used data on prevalence of mutated strains in Tokyo as causative strains. If the vaccine history and causative strains of the patients themselves were available, the results might be different.

Third, although we compared mortality among an administrated group and an unadministered group based on the estimated propensity score at the first step, we also control demographical condition and/or underlying diseases, in addition to epidemiological situations for mortality. That remains as a challenge for our future research.

Fourth, the drugs considered for this study were not always administered to the most severely ill patients. For example, antibody cocktails were administered to patients with less–severe illness. However, to simplify the analysis used for this study, we did not incorporate the combination of drugs or change from one considered drug to another considered drug. That choice might bias the results to some degree. Such a process or pattern of drug administration might be important to evaluate drug effectiveness.

## Conclusion

The obtained results showed that antibody cocktails for all older patients and all inpatients with moderate severity, tocilizumab for severely ill younger inpatients, and baricitinib for younger inpatients with moderate illness severity might contribute to saving life. In the latter two drugs, tocilizumab and baricitinib, some treatment effects suggested a reduction of saving life for all or older inpatients. We infer that the effects of these drugs were mixed. In fact, remdesivir and dexamethasone might not contribute to saving life. However, more information, including test results, is needed for better matching and achievement of definitive conclusions.

## Data Availability

The data in this study was provided from the National Hospital Organization (NHO), but the availability of these data was restricted because of privacy policy of NHO. This data was used under license for this study and were therefore not available to the public. Authors were permitted to use this data by the Ethical Committee of Mie Hospital and NHO Committee. Figshare: Drug effectiveness for COVID-19 inpatients inferred from Japanese medical claim data using propensity score matching_supplement data.csv https://doi.org/10.6084/m9.figshare.22102016.v1. ^19^ Data are available under the terms of the Creative Commons Attribution 4.0 International license (CC-BY 4.0).
