# Drug effectiveness for COVID-19 inpatients inferred from Japanese medical claim data using propensity score matching

**DOI:** 10.12688/f1000research.131102.2

**Published:** 2024-01-22

**Authors:** Shingo Mitsushima, Hiromasa Horiguchi, Kiyosu Taniguchi

**Affiliations:** 1 Center for Field Epidemic Intelligence, Research and Professional Development, National Institute of Infectious Diseases, Shinjuku-ku, Tokyo, 1620052, Japan; 2 Department of Clinical Data Management and Research, Clinical Research Center, National Hospital Organization, Meguro-ku, Tokyo, 1528621, Japan; 3 Research Director, The Tokyo Foundation for Policy Research, Minato-ku, Tokyo, 1066234, Japan; 4 General Director, National Hospital Organization Mie National Hospital, Tsu, Mie, 5140125, Japan

**Keywords:** COVID-19, mutated strain, underlying diseases, antibody cocktail, antiviral drug, mortality, steroid and anti-inflammatory drug

## Abstract

**Background:**

Earlier studies and clinical trials of Coronavirus 2019 (COVID-19) showed that drugs such as antiviral drugs, antibody cocktails, and steroids and anti-inflammatory drugs can prevent severe outcomes and death.

**Methods:**

Observational data in Japan assess drug effectiveness against COVID-19. We applied the average treatment effect model, particularly propensity scoring, which can treat the choice of administered drug as if administration were randomly assigned to inpatients. Data of the Medical Information Analysis Databank, operated by National Hospital Organization in Japan, were used. The outcome was defined as mortality. Subjects were all inpatients, inpatients with oxygen administration, and inpatients using respiratory ventilation, classified by three age classes: all ages, 65 years old or older, and younger than 65 years old. Information about demographic characteristics, underlying disease, administered drug, the proportions of Alpha, Beta and Omicron variant strains, and vaccine coverage were used as explanatory variables for logistic regression.

**Results:**

Estimated results indicated that only one antibody cocktail (sotrovimab, casirivimab and imdevimab) was associated with raising the probability of survival consistently and significantly. By contrast, other drugs, an antiviral drug (remdesivir), a steroid (dexamethasone), and an anti-inflammatory drug (baricitinib and tocilizumab) were related to reduce the probability of survival. However, propensity score matching method might engender biased results because of a lack of data such as detailed information related to intervention and potential confounders. Therefore, the effectiveness of some drugs might not be evaluated properly in this study.

**Conclusions:**

Results indicate high likelihood that antibody cocktails were consistently associated with high probability of survival, although low likelihood was found for other drugs for older patients with mild to severe severity and all age patients with moderate severity. Further study is necessary in light of the lack of available data.

## Introduction

Coronavirus disease 2019 (COVID-19) is caused by severe acute respiratory syndrome coronavirus 2 (SARS-CoV-2). COVID-19 caused pandemic throughout the world from December 2019. In Japan, the first case was detected in January 2020; it then spread across the country. As characteristics of COVID-19 were revealed and treatments developed, the mortality rate attributable to COVID-19 has declined. ^1^

Earlier studies and clinical trials have shown that drugs used against COVID-19, such as antiviral drugs (remdesivir), ^2^ antibody cocktails (casirivimab/imdevimab and sotrovimab), ^3^^,^^4^ steroids (dexamethasone), ^5^ anti-inflammatory drugs (baricitinib and tocilizumab), ^6^^–^^8^ statins, ^9^^,^^10^ RNA-dependent RNA polymerase inhibitor (molnupiravir), ^11^^,^^12^ and protease inhibitor (nirmatrelvir/ritonavir), ^11^^,^^13^ are expected to prevent severe COVID-19 outcomes and death.

The National Hospital Organization (NHO) in Japan, an organization of regional core hospitals accounting for about 3.4% of all beds in Japan, ^14^ provides a database of medical claims from 60 representative NHO hospitals: the Medical Information Analysis Databank (MIA). NHO is one of the largest hospital organizations. Each prefecture has one or more NHO hospitals. MIA includes accumulated data of medical insurance claims for outpatients and inpatients. It includes inpatients’ demographic information, underlying diseases, medical interventions including oxygen administration, use of respiratory ventilation, administration of drugs, and outcomes such as discharge or death. ^15^

We evaluated the top five most commonly used drugs in MIA: remdesivir, dexamethasone, baricitinib, tocilizumab, and antibody cocktails (casirivimab/imdevimab and sotrovimab). In earlier studies, all these drugs were associated with improved survival or reduced the risk of disease progression, but antibody cocktails were effective only for mildly ill patients; dexamethasone was only effective for moderately to severely ill patients. ^2^^–^^8^ We did not divide the antibody cocktails sotrovimab and casirivimab/imdevimab because the numbers of cases with antibody cocktails were insufficiently large. We considered the overall effects of antibody cocktail treatments.

Whether a drug is administered or not depends on the patient condition. In other words, drug administration is probably not a random assignment. Generally speaking, patients who have a higher likelihood of developing an illness have a higher probability of being administered drugs, which implies, because of selection bias in the observational data, a negative association between drug administration and outcomes. Non-random choice of drug administration must be considered for drug effectiveness estimation. Propensity score matching might resolve this difficulty statistically but not experimentally. ^16^ Propensity score matching is used widely in social sciences to evaluate programs: participants choose to join a program spontaneously, such as a job training program or an unemployment payment program. However, in medical sciences, a researcher can perform experiments and thereafter delete selection bias in the choice of subjects. Nevertheless, such experiments are expensive and require a longer period. These limitations cause the number of subjects in experiments to be constrained. Moreover, outcomes tend to be evaluated with less severity. ^17^^,^^18^ Moreover, after approval, experiments with random assignment are expected to be difficult to conduct in the real world because of ethical considerations: some patients might be unable to receive a potentially effective drug. Therefore, pseudo-experimental method should be applied to evaluate drug effectiveness in the real world. An average treatment effect model including propensity matching was applied to create these situations. This procedure predicts the likelihood of receiving drugs initially. Thereafter, outcomes of a drug-administered group and a drug not-administered group are compared. The members of those groups have an almost equal likelihood of having been administered some drugs. We then examined drug effectiveness using this method.

The objective of this study was evaluating the “real-world effectiveness of drugs against COVID-19 in Japan.” “Real-world effectiveness of drugs” means the effectiveness of drugs considering several factors including population characteristics, examinations, vaccinations, and healthcare system such as hospitalization and treatment protocols. Therefore, the results of this study might not be applicable to other countries.

## Method

### Data sources

This study used MIA for confirmed inpatients: age, sex, underlying diseases, hospitalization date, administered drug, outcome and whether they received oxygen therapy and/or ventilation. We created an overview of inpatients in MIA and patients in Japan based on National data and MIA data.

We also used data for vaccine administration published by the Cabinet Secretariat. Moreover, prevalence of Alpha, Delta and Omicron variant strains were referred from a monitoring meeting in Tokyo because MIA included no information about a patient’s vaccine status or sublineage in SARS-CoV-2.

The study period was January 2020 through March 2022, using data recorded as of May 2022. This study area was the entirety of Japan.

### Definitions of variables

We defined demographical conditions as age and sex, underlying diseases as cancer (C00–C90 in ICD10), asthma (J45), chronic obstructive pulmonary disease (COPD) (J44), hypertension (HT) (I10), heart failure (HF) (I50), and diabetes mellitus (DM) (E10). We examined the effects of an antiviral drug (remdesivir), ^2^ an antibody cocktail (sotrovimab and casirivimab/imdevimab), ^3^^,^^4^ a steroid (dexamethasone), ^5^ and anti-inflammatory drugs (baricitinib and tocilizumab), ^6^^–^^8^ which were proven effective against COVID-19 by earlier studies. We assumed vaccination coverage as the rate of the second dose of vaccine received two weeks prior by age class, as younger than 65 years old, and 65 years old or older. Mutated strains, Alpha, Delta, and Omicron variant strains, were measured by percentage at one week before admission. The Alpha, Delta, and Omicron variant strains were defined as the respective proportions of Alpha, Delta, and Omicron variant strains. Omicron included BA.2 or a later sublineage. Alternatively, we used a dummy variable during the 4th–6th wave instead of the proportion of the mutated strains as an explanatory variable, to check robustness. By this specification, the Alpha variant strain emerged and then dominated in the 4th wave, defined as from 1 March through 20 June 2021. Similarly, the Delta variant strain emerged and then dominated in the 5th wave, defined as 21 June through 21 November 2021. Omicron BA.1 strain emerged and then dominated from the 6th wave, defined as 22 November 2021 to the end of the study period. Death during hospitalization was defined as the outcome.

### Subjects

Subjects were all inpatients confirmed from MIA data as having SARS-CoV-2. For this study, mild symptoms were defined as a patient who did not require medical intervention such as oxygen therapy or respiratory ventilation, moderate symptoms were defined as a patient who required oxygen administration, and severe symptoms were defined as a patient who required respiratory ventilation.

In Japan, during the Delta variant epidemic, patients with mild severity were not admitted to hospitals because medical resources such as hospital beds had reached full capacity, although they could have been hospitalized before the period of the Delta variant epidemic. Aside from purely medical criteria, the criteria of hospitalization for asymptomatic patients or patients with mild symptoms who did not require oxygen therapy were probably affected strongly by medical resource scarcity or social situations such as support for their staying at home and recuperation at home. For that reason, we also limited our study to subjects who were expected to be inpatients with oxygen therapy or respiratory ventilation.

### Statistical analysis

We conducted estimates separately by drug type: remdesivir, antibody cocktail (sotrovimab, casirivimab and imdevimab), dexamethasone, baricitinib, and tocilizumab.

The first step to assess whether the patient was administered a type of drug or not was performed through logistic regression on their age, sex, underlying diseases, pharmaceutical therapy, vaccine coverage, and prevalence in the mutated strains as explanatory variables. The second step was comparison of outcomes among administered patients and non-administered patients with almost identical likelihood to that of the prediction done in the first step.

All statistical analyses were conducted using software (Stata SE 17.0; Stata Corp.). We inferred significance at the 5% level.

### Ethical considerations

Individual informed consent was not required to conduct this study because the dataset was provided as anonymized data by NHO. ^15^ This study was approved by the Ethics Committee of Mie Hospital (Approval No. 2020-89) on 23 October 2020. Specific permission to use MIA data was obtained from the NHO (Registration No. 1201003) on 1 December 2020.

## Results

**Figure 1.**
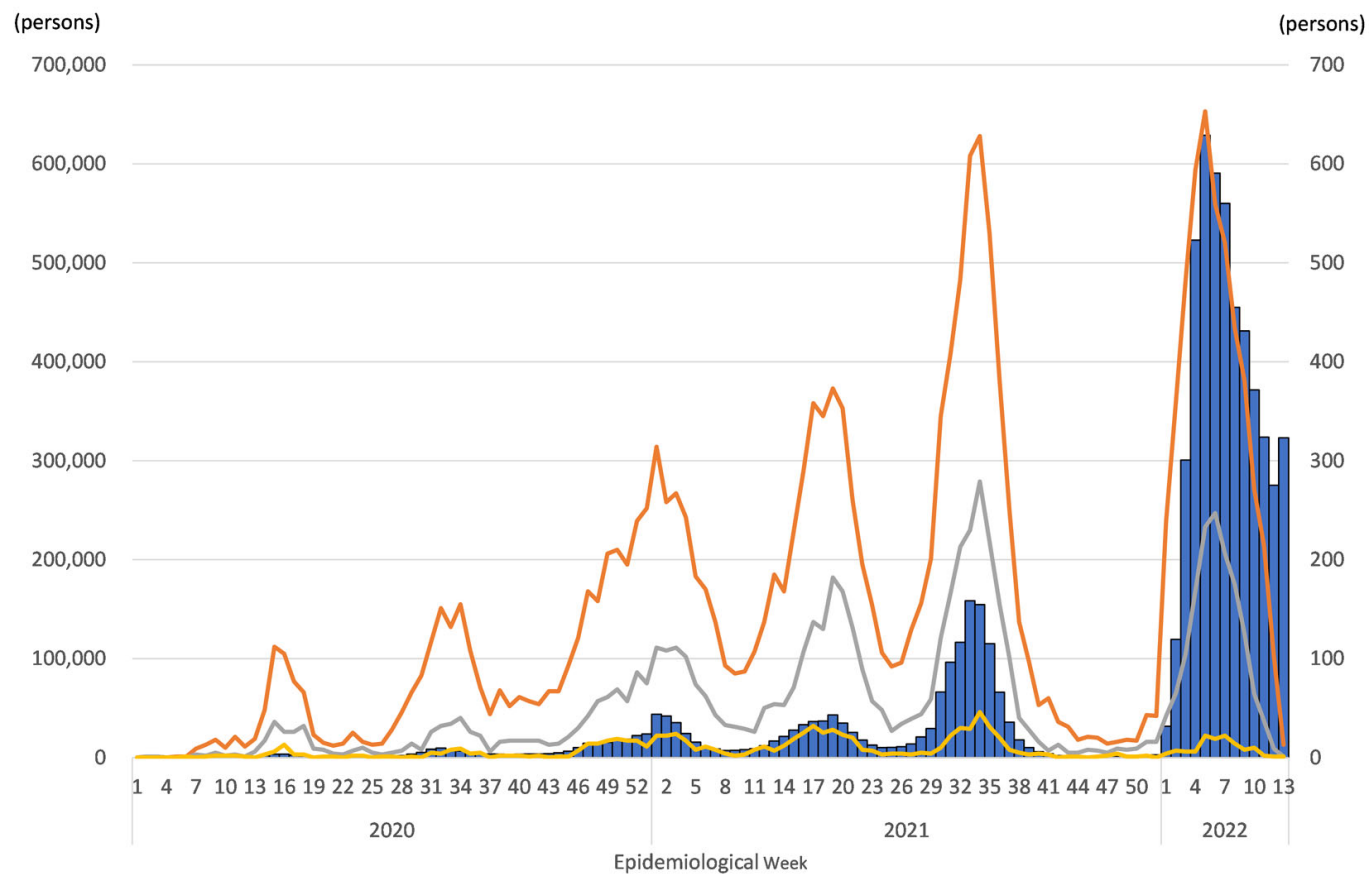
COVID-19 patients of National data and COVID-19 inpatients of MIA data. Notes: Bars show the number of COVID-19 patients in National data (left scale). Orange, gray, and yellow lines respectively show the numbers of COVID-19 inpatients in MIA, COVID-19 inpatients with oxygen therapy, and COVID-19 inpatients with respiratory ventilation (right scale).

**Figure 2.**
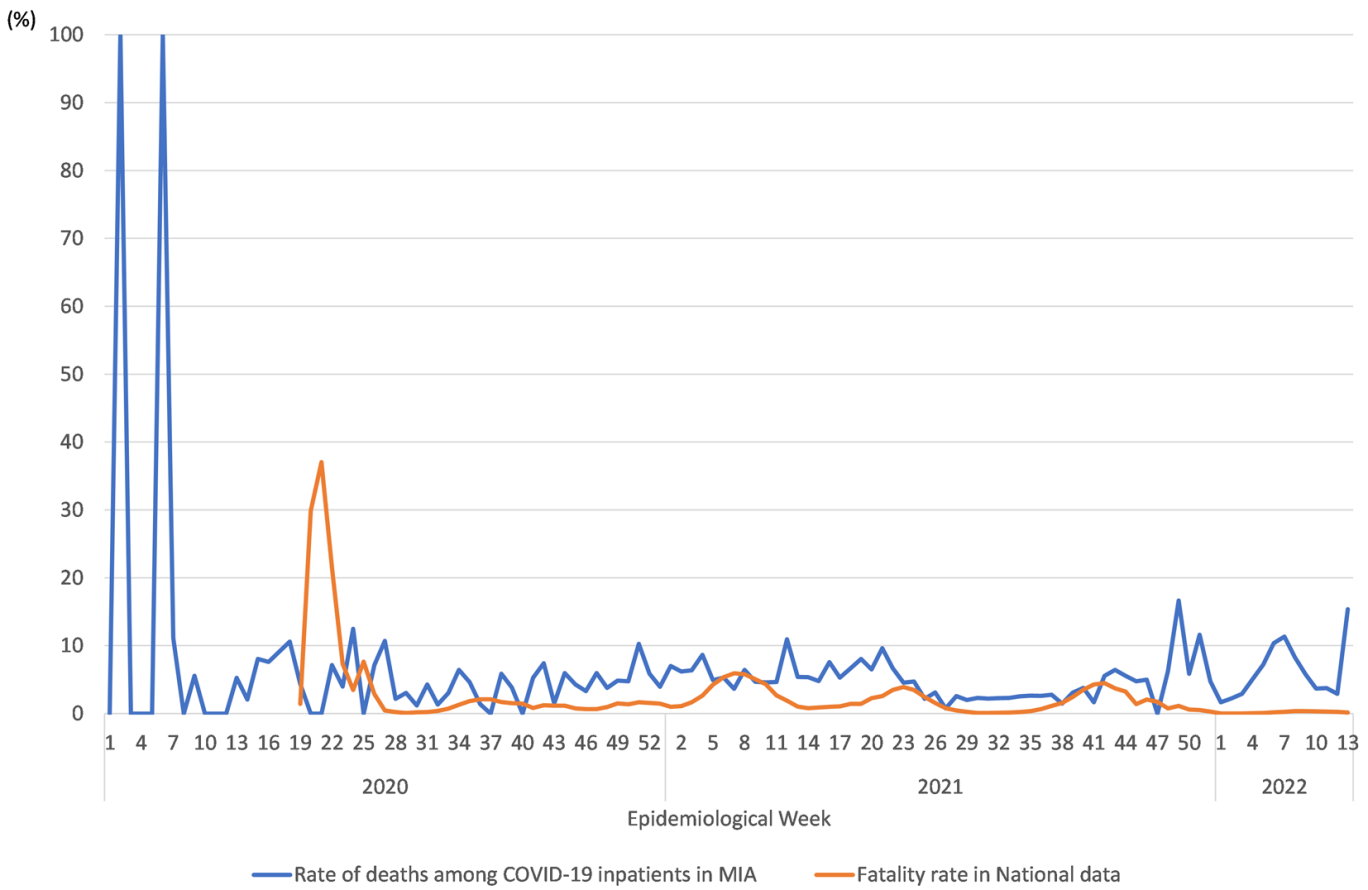
Fatality rate for National data and Rate of deaths among COVID-19 inpatients in MIA. Notes: Blue and orange lines respectively denote the rate of deaths among COVID-19 inpatients in MIA and the COVID-19 fatality rate in National data. Data for COVID-19 patients and deaths in 0–18 week in National data were unavailable.

Table 1 shows the number of COVID-19 patients from National data and the numbers of inpatients, inpatients with oxygen therapy, and inpatients with respiratory ventilation in MIA data. These data show similar trends. Most notably, several surges occurred from January 2020 through March 2022, as shown in Figure 1. Figure 2 presents the COVID-19 fatality rate in National data and the rate of deaths among COVID-19 inpatients in MIA data. These data were less than 10%. They followed a similar trend. A supplementary table (see *Extended data* ^21^), presents a summary of the estimation results obtained for the first step logistic regression for drug administration. Table 1 presents estimation results obtained for all inpatients, inpatients with oxygen therapy, and for inpatients who used a respiratory ventilation in three age classes. Results show 24 significance estimators of 90 estimators in all, but 20 estimators were positive, which indicates that drug administration was associated significantly with low probability of survival.

**Table 1.** Estimation results of propensity score matching method.

Age class	All		65 years old and older		Younger than 65 years old	
	difference	*p*-value	difference	*p*-value	difference	*p*-value
Remdesivir						
Proportion						
All	0.034	0.000	0.064	0.000	0.019	0.068
Oxygen administration	0.015	0.315	0.045	0.056	0.013	0.311
Respiratory ventilator used	0.012	0.768	0.127	0.080	N.A.	N.A.
Period						
All	0.031	0.000	0.067	0.004	0.010	0.126
Oxygen administration	0.012	0.348	0.044	0.087	0.011	0.337
Respiratory ventilator used	-0.006	0.893	0.047	0.477	N.A.	N.A.
Dexamethasone						
Proportion						
All	0.027	0.000	0.079	0.000	0.003	0.253
Oxygen administration	0.020	0.013	0.052	0.000	0.006	0.234
Respiratory ventilator used	-0.021	0.548	-0.055	0.296	0.044	0.319
Period						
All	0.029	0.000	0.076	0.000	0.004	0.119
Oxygen administration	0.014	0.090	0.038	0.011	0.006	0.131
Vent	-0.051	0.158	-0.083	0.125	0.008	0.857
Tocilizumab						
Proportion						
All	0.091	0.000	0.214	0.000	0.019	0.057
Oxygen administration	0.079	0.001	0.126	0.021	N.A.	N.A.
Respiratory ventilator used	0.117	0.227	N.A.	N.A.	-0.077	0.030
Period						
All	0.103	0.000	0.223	0.000	0.025	0.030
Oxygen administration	0.086	0.001	0.126	0.020	N.A.	N.A.
Respiratory ventilator used	0.090	0.375	N.A.	N.A.	-0.062	0.079
Baricitinib						
Proportion						
All	0.023	0.100	0.411	0.114	N.A.	N.A.
Oxygen administration	-0.016	0.220	-0.033	0.900	-0.008	0.023
Respiratory ventilator used	-0.149	0.586	0.384	0.000	N.A.	N.A.
Period						
All	N.A.	N.A.	N.A.	N.A.	N.A.	N.A.
Oxygen administration	N.A.	N.A.	N.A.	N.A.	N.A.	N.A.
Respiratory ventilator used	N.A.	N.A.	N.A.	N.A.	N.A.	N.A.
Sotrovimab or casirivimab/imdevimab						
Proportion						
All	N.A.	N.A.	-0.105	0.000	N.A.	N.A.
Oxygen administration	-0.085	0.000	N.A.	N.A.	N.A.	N.A.
Respiratory ventilator used	N.A.	N.A.	N.A.	N.A.	N.A.	N.A.
Period	N.A.	N.A.	N.A.	N.A.	N.A.	N.A.
All	N.A.	N.A.	N.A.	N.A.	N.A.	N.A.
Oxygen administration	N.A.	N.A.	N.A.	N.A.	N.A.	N.A.
Respiratory ventilator used	N.A.	N.A.	N.A.	N.A.	N.A.	N.A.

**Abbreviations:** N.A., not available.

**Notes**: Yellow denotes significance.

Four estimators indicating a drug as probably related to high probability of survival were the following: tocilizumab among younger inpatients with ventilation; baricitinib among younger inpatients with oxygen therapy; and antibody cocktails among all older inpatients and among all inpatients with oxygen therapy. Of the three drugs, only the antibody cocktail had no positive difference, but association with an increased probability of survival. Some treatment effects of tocilizumab and baricitinib were positive, especially for all older inpatients or for older inpatients with moderate severity. Therefore, the results obtained for these two drugs were mixed.

## Discussion

We estimated drug effectiveness separately. Results showed that antibody cocktails might contribute to survival. The estimated results for tocilizumab and baricitinib were mixed, but these drugs were presumed to provide some probability of survival among younger but severely ill patients. Nevertheless, we were unable to find any evidence that remdesivir or dexamethasone contributes to survival at all. We must assess the drugs’ effectiveness after approval using observational data because factors such as the emergence of mutated strains, vaccination, and development of therapies can affect drug efficacy.

These counterintuitive findings, which are inconsistent with results of earlier studies indicating the effectiveness of remdesivir and dexamethasone, might be attributable to several limitations. First, worse matching at the first step could engender biased results. ^2^^,^^5^ The propensity score matching method could engender biased results because of lack of data such as detailed information related to intervention and potential confounders. This bias results from insufficient data such as potential confounders we did not measure and from a lack of detailed information related to interventions (high flow nasal canula or extracorporeal membrane oxygenation) and underlying diseases recognized as risk factors for severe COVID-19 (obesity or immunosuppression). Second, we examined the data, particularly using Japanese vaccine coverage instead of the vaccination history of the patients. We also used data of the prevalence of mutated strains in Tokyo as causative strains. If the vaccine history and causative strains of the patients themselves were available, then the results might be different. Third, this study was an observational study that was able to show only association between considered drugs and death, not necessarily a causal association. Fourth, endogeneity might not be controlled well in decisions to use drugs. Drugs to be administered against COVID-19 depend on the severity of illness of the patient. For instance, an earlier report described dexamethasone as effective for moderate or severe COVID-19, but as ineffective for mild cases. ^5^ Actually, more than antibody cocktails, ^3^^,^^4^ remdesivir ^2^ and dexamethasone ^5^ tend to be administered more for severely ill patients. Finally, to simplify the analysis used for this study, we did not incorporate the combination of drugs or change from one considered drug to another considered drug. That choice might bias the results to some degree. Such a process or pattern of drug administration might be important to evaluate drug effectiveness. Intercorrelation among drugs should be incorporated into the estimation model in future studies.

In our results, only antibody cocktails were found to be associated with high probability of survival. An earlier study indicated that casirivimab/imdevimab, an antibody cocktail, lowered the viral load. ^3^ Typically, these antibody cocktails were given to patients with mild severity, which means that they were administered before a marked increase in viral load. This pattern of administration might be one reason why antibody cocktails were effective for survival. The antibody cocktail results included many “not available” entries, although drug effectiveness was confirmed in well-matched cases. The approval of antibody cocktails was late. The number of prescriptions was simply low, which might affect many cases for which “not available” was reported. In Japan, remdesivir, baricitinib, casirivimab/imdevimab, sotrovimab, and tocilizumab were approved for treatment of COVID-19, respectively, in May 2020, April 2021, July 2021, September 2021, and January 2022. ^19^ Data accumulation might resolve that shortcoming.

We used MIA data of inpatients to verify drug effectiveness. In our earlier study, although we confirmed the representativeness of fatality cases using MIA data, the data on the numbers of patients with oxygen therapy and respiratory ventilation in Japan have not been published, yet. ^20^ We have not verified the representativeness of patients with oxygen therapy and respiratory ventilation. Moreover, because MIA is a database of medical claims, data of the prior few weeks might change during a few months. For this study, we collected and analyzed data from January 2020 through the end of March 2022, recorded as of May 2022. If the study period was extended, the estimation results might change over time because the accumulated data engender more accurate results. Emerging new mutated strains, immunization, and development of new therapies might affect the results. Inclusion of additional data and extending the study period remain as challenges for future research.

## Conclusion

The obtained results demonstrated that antibody cocktails for all older patients and all inpatients with moderate severity, tocilizumab for severely ill younger inpatients, and baricitinib for younger inpatients with moderate severity might contribute to survival. For the latter two drugs, tocilizumab and baricitinib, some treatment effects suggest a reduction of survival for all or older inpatients. We infer that the effects of these drugs were mixed. In fact, remdesivir and dexamethasone might not contribute to survival. However, more information, including test results, is needed for better matching and for the achievement of definitive conclusions.

## Data Availability

The data used for this study were provided by the National Hospital Organization (NHO), but the availability of these data was restricted because of privacy policies of NHO. These data were used under license for this study and are therefore not available to the public. Authors were permitted to use these data by the Ethical Committee of Mie Hospital and NHO Committee. Figshare: Drug effectiveness for COVID-19 inpatients inferred from Japanese medical claim data using propensity score matching_supplement data.csv https://doi.org/10.6084/m9.figshare.22102016.v1. ^21^ Data are available under the terms of the Creative Commons Attribution 4.0 International license (CC-BY 4.0).
