# Supplementary material for: Drug effectiveness for COVID-19 inpatients inferred from Japanese medical claim data using propensity score matching
Source: F1000Res. 2024 Jan 22;12:398. Originally published 2023 Apr 13. [Version 2] doi: 10.12688/f1000research.131102.2 (PMC11299052; doi:10.12688/f1000research.131102.2)
Supplement: Peer review history

# Reviewer report: Reviewer response for version 2

**DOI:** 10.5256/f1000research.161283.r305927

**Published:** 2024-08-01

**Authors:** Junko Kurita

**Affiliations:** 1 Department of Nursing, Faculty of Sports & Health Science, Daitobunka University, Higashimatsuyama-shi, Japan

"Drug effectiveness for COVID-19 inpatients inferred from Japanese medical claim data  using propensity score matching" was an excellent study investigating drug effectiveness in the real world. Even though it was approved through rct, effectiveness in the real world might be different from rct. So as to realize an experimental situation in the observational clinical data, they used propensity score matching procedure.

 I congratulate the author's clear analysis in this field. However, I have some minor points to revise.

**Minor points:**

 1) Figure 2 shows that there were COVID-19 patients and died in the first or second week, 2020, in MIA dara.  I understand that the initial COVID-19 patient was confirmed on January 15, 2020 and thus the third or fourth week of 2020. Was the initial patient in MIA true ? Or had these patients administered in the first week of 2020 before COVID-19 infection, but been infected in COVID-19 in the hospital much later and died ?

 2) Because the study period was from January 2020, was the period before approve mentor recommendation in guideline for COVID-19 included in the study period ? If so, it might cause some bias to the results. It might be just a comparison before and after approvement.

 3) Though the yellow marks were very clear, but it might not be the usual presentation.

 I recommended to remove yellow marks.

 4) "Proportion" or "period" in Table 1 were unclear. Could you add words in Table and/or add more explanation about them in legend.

Is the work clearly and accurately presented and does it cite the current literature?

Yes

If applicable, is the statistical analysis and its interpretation appropriate?

Yes

Are all the source data underlying the results available to ensure full reproducibility?

Yes

Is the study design appropriate and is the work technically sound?

Yes

Are the conclusions drawn adequately supported by the results?

Yes

Are sufficient details of methods and analysis provided to allow replication by others?

Yes

Reviewer Expertise:

statistical and/or mathematical analysis for medical data

I confirm that I have read this submission and believe that I have an appropriate level of expertise to confirm that it is of an acceptable scientific standard.

# Reviewer report: Reviewer response for version 2

**DOI:** 10.5256/f1000research.161283.r239585

**Published:** 2024-04-11

**Authors:** Ramesh Kandimalla

**Affiliations:** 1 Biochemistry, Kakatiya Medical College, Warangal, Telengana, India

All the comments were addressed appropriately. No more additional comments for me

Is the work clearly and accurately presented and does it cite the current literature?

Partly

If applicable, is the statistical analysis and its interpretation appropriate?

Yes

Are all the source data underlying the results available to ensure full reproducibility?

Yes

Is the study design appropriate and is the work technically sound?

Partly

Are the conclusions drawn adequately supported by the results?

Partly

Are sufficient details of methods and analysis provided to allow replication by others?

Partly

Reviewer Expertise:

COVID-19, Diabetes, Alzheimer's, Mitochondria, Carbon dots, Nanospheres

I confirm that I have read this submission and believe that I have an appropriate level of expertise to confirm that it is of an acceptable scientific standard.

# Reviewer report: Reviewer response for version 1

**DOI:** 10.5256/f1000research.143911.r214688

**Published:** 2023-11-10

**Authors:** Ramesh Kandimalla

**Affiliations:** 1 Biochemistry, Kakatiya Medical College, Warangal, Telengana, India

1. The methods section is detailed, but it could benefit from additional clarity in explaining the rationale behind using propensity scoring and the choice of variables in logistic regression. This could help readers better understand the study design.
2. It would be helpful to provide more information about the reliability and representativeness of the observational data from Japan. Mentioning any limitations or potential biases in the data source would enhance the transparency of the study.
3. The abstract implies causal relationships between drug administration and outcomes, but the observational nature of the study might only establish correlations. It's essential to acknowledge the limitations in inferring causation from observational data.
4. The abstract mentions that the logistic regression might have "insufficient explanatory power." It would be beneficial to elaborate on this limitation and discuss potential sources of bias or confounding that could affect the study's validity.
5.  When discussing the drugs, providing a brief rationale for why certain drugs might be effective or ineffective could enhance the comprehensibility of the results.
6. The mention of the Omicron variant is timely, but given the evolving nature of the pandemic, it's important to note the potential for changes in outcomes as new variants emerge. This could affect the generalizability of the findings.
7. The abstract could include a brief section discussing the limitations of the study, such as potential confounders not accounted for in the analysis or any challenges in accurately measuring certain variables.
8. The use of phrases like "in Japan" and "against COVID-19" is repeated frequently. Adding variety to the language could enhance the readability of the introduction.
9. The timeline of events related to COVID-19 in Japan could be presented more clearly. For example, mentioning the initial surge in cases, the development of treatments, and the subsequent decline in mortality rate in a more chronological order could improve the flow.
10. The introduction references earlier studies and clinical trials but doesn't provide specific citations for the mentioned data or findings. Including citations within the text could enhance the credibility of the information presented.
11. While the introduction mentions the decline in the mortality rate due to COVID-19 in Japan, it might be beneficial to include specific statistics or percentages to quantify this change and emphasize the significance.
12. The term "real-world effectiveness" is used, but a brief explanation or definition could be included to clarify its meaning for readers who may not be familiar with the term.
13. The limitations of the MIA data, such as the absence of vaccination history or causative strain information, are mentioned but could be discussed more explicitly. Acknowledging these limitations upfront adds transparency to the study.
14. The transition from providing background information to discussing the methods is somewhat abrupt. Adding a sentence or two to smoothly transition into the methods section could improve the overall flow of the introduction.
15. While the introduction mentions propensity score matching, a brief explanation of how it works and its purpose in addressing selection bias could enhance reader understanding.
16. The introduction could touch upon the external validity of the study, acknowledging that findings may not be universally applicable due to variations in healthcare systems, populations, or treatment protocols.
17. The introduction could explicitly state the research question or hypothesis being addressed by the study to guide readers in understanding the study's purpose from the outset.
18. The text repeats certain points, such as the mention of "propensity score" and "saving life," multiple times. Reducing redundancy could improve the overall clarity and conciseness of the text.
19.  Some sentences are quite complex, which might make it challenging for readers to follow the arguments. Simplifying the language without compromising precision could enhance comprehension.
20. The text occasionally uses language that implies causation, such as "might contribute to saving life." It's important to be cautious about making causal claims based on observational data. Acknowledging the limitations in establishing causation would strengthen the study's validity.
21. The text mentions "not available" entries in the results for antibody cocktails, attributing it to a small sample size. Providing a more explicit explanation of why certain data is missing and its potential impact on the results would enhance transparency.
22. The limitations section is placed towards the end of the text. Moving it closer to the beginning or integrating limitations as they become relevant in the discussion could improve the flow and help readers contextualize the findings.
23. The use of terms like "severely ill," "moderate severity," and "less–severe illness" could benefit from clearer operational definitions or references to specific criteria used for classification.
24. The discussion section covers a range of topics, including limitations and future research, without clear organization. Structuring the discussion into subsections could make it more reader-friendly.

Is the work clearly and accurately presented and does it cite the current literature?

Partly

If applicable, is the statistical analysis and its interpretation appropriate?

Yes

Are all the source data underlying the results available to ensure full reproducibility?

Yes

Is the study design appropriate and is the work technically sound?

Partly

Are the conclusions drawn adequately supported by the results?

Partly

Are sufficient details of methods and analysis provided to allow replication by others?

Partly

Reviewer Expertise:

COVID-19, Diabetes, Alzheimer's, Mitochondria, Carbon dots, Nanospheres

I confirm that I have read this submission and believe that I have an appropriate level of expertise to confirm that it is of an acceptable scientific standard, however I have significant reservations, as outlined above.

# Response

**Published:** 2024-01-10

**Authors:** Shingo Mitsushima

**Affiliations:** National Institute of Infectious Diseases, Japan, Japan

1. The methods section is detailed, but it could benefit from additional clarity in explaining the rationale behind using propensity scoring and the choice of variables in logistic regression. This could help readers better understand the study design.

 Response: We added the following sentence to Introduction.

 “Moreover, after approval, experiments with random assignment are expected to be difficult to conduct in the real world because of ethical considerations: some patients might be unable to receive a potentially effective drug. Therefore, pseudo-experimental method should be applied to evaluate drug effectiveness in the real world.”

 2. It would be helpful to provide more information about the reliability and representativeness of the observational data from Japan. Mentioning any limitations or potential biases in the data source would enhance the transparency of the study.

 Response: We added the following sentence to Introduction.

 “In our earlier study, although we confirmed the representativeness of fatality cases using MIA data, the data on the numbers of patients with oxygen therapy and respiratory ventilation in Japan have not been published, yet. We have not verified the representativeness of patients with oxygen therapy and respiratory ventilation.”

 3. The abstract implies causal relationships between drug administration and outcomes, but the observational nature of the study might only establish correlations. It's essential to acknowledge the limitations in inferring causation from observational data.

 Response: We revised sentences in Abstract as below.

 OLD: “raised the probability to save life” “contribute to saving life”

 NEW: “was associated with raising the probability of survival” “were related to reduced probability of survival”

 In addition, we added the following sentence to Discussion.

 “this study was an observational study that was able to show only association between considered drugs and death, not necessarily a causal association.”

 4. The abstract mentions that the logistic regression might have "insufficient explanatory power." It would be beneficial to elaborate on this limitation and discuss potential sources of bias or confounding that could affect the study's validity.

 Response: We added the following sentence to Abstract.

  “However, propensity score matching method might engender biased results because of a lack of data such as detailed information related to intervention and potential confounders. Therefore, the effectiveness of some drugs might not be evaluated properly in this study.”

 5. When discussing the drugs, providing a brief rationale for why certain drugs might be effective or ineffective could enhance the comprehensibility of the results.

 Response: We added the following sentences to Discussion.

 “An earlier study indicated that casirivimab/imdevimab, an antibody cocktail, lowered the viral load. Typically, these antibody cocktails were given to patients with mild severity, which means that they were administered before a marked increase in viral load. This pattern of administration might be one reason why antibody cocktails were effective for survival.”

 6. The mention of the Omicron variant is timely, but given the evolving nature of the pandemic, it's important to note the potential for changes in outcomes as new variants emerge. This could affect the generalizability of the findings.

 Response: We added the following sentences to Discussion.

 “If the study period was extended, the estimation results might change over time because the accumulated data engender more accurate results. Emerging new mutated strains, immunization, and development of new therapies might affect the results.”

 7. The abstract could include a brief section discussing the limitations of the study, such as potential confounders not accounted for in the analysis or any challenges in accurately measuring certain variables.

 Response:

 We added the following sentences to Abstract.

 “However, propensity score matching method might engender biased results because of a lack of data such as detailed information related to intervention and potential confounders. Therefore, the effectiveness of some drugs might not be evaluated properly in this study.”

 8. The use of phrases like "in Japan" and "against COVID-19" is repeated frequently. Adding variety to the language could enhance the readability of the introduction.

 Response: We replaced some words “Japan” and “COVID-19” with alternative words.

 9. The timeline of events related to COVID-19 in Japan could be presented more clearly. For example, mentioning the initial surge in cases, the development of treatments, and the subsequent decline in mortality rate in a more chronological order could improve the flow.

 Response: We added Figure 1 and Figure 2 to show the timeline of COVID-19 events. We also added the following sentences to Result.

 “Table 1 shows the number of COVID-19 patients from National data and the numbers of inpatients, inpatients with oxygen therapy, and inpatients with respiratory ventilation in MIA data. These data show similar trends. Most notably, several surges occurred from January 2020 through March 2022, as shown in Figure 1. Figure 2 presents the COVID-19 fatality rate in National data and the rate of deaths among COVID-19 inpatients in MIA data. These data were less than 10%. They followed a similar trend.”

 10. The introduction references earlier studies and clinical trials but doesn't provide specific citations for the mentioned data or findings. Including citations within the text could enhance the credibility of the information presented.

 Response: We added the following sentence to Introduction.

 “In earlier studies, all these drugs were associated with improved survival or reduced the risk of disease progression, but antibody cocktails were effective only for mildly ill patients; dexamethasone was only effective for moderately to severely ill patients.”

 11. While the introduction mentions the decline in the mortality rate due to COVID-19 in Japan, it might be beneficial to include specific statistics or percentages to quantify this change and emphasize the significance.

 Response: We drew Figure 2, which shows fatality rates in National data and rate of deaths among COVID-19 inpatients with specific percentage.

 12. The term "real-world effectiveness" is used, but a brief explanation or definition could be included to clarify its meaning for readers who may not be familiar with the term.

 Response: We added the following sentences to Introduction.

 “The objective of this study was evaluating the “real-world effectiveness of drugs against COVID-19 in Japan.” “Real-world effectiveness of drugs” means the effectiveness of drugs considering several factors including population characteristics, examinations, vaccinations, and healthcare system such as hospitalization and treatment protocols.”

 13. The limitations of the MIA data, such as the absence of vaccination history or causative strain information, are mentioned but could be discussed more explicitly. Acknowledging these limitations upfront adds transparency to the study.

 Response: We added the following sentences about the limitation of vaccination history and causative strain to Discussion.

 “Second, we examined the data, particularly using Japanese vaccine coverage instead of the vaccination history of the patients. We also used data of the prevalence of mutated strains in Tokyo as causative strains. If the vaccine history and causative strains of the patients themselves were available, then the results might be different.”

 We also added the sentences about the limitation of MIA data to Discussion.

 “In our earlier study, although we confirmed the representativeness of fatality cases using MIA data, the data on the numbers of patients with oxygen therapy and respiratory ventilation in Japan have not been published, yet. We have not verified the representativeness of patients with oxygen therapy and respiratory ventilation. Moreover, because MIA is a database of medical claims, data of the prior few weeks might change during a few months. For this study, we collected and analyzed data from January 2020 through the end of March 2022, recorded as of May 2022. If the study period was extended, the estimation results might change over time because the accumulated data engender more accurate results. Emerging new mutated strains, immunization, and development of new therapies might affect the results.”

 14. The transition from providing background information to discussing the methods is somewhat abrupt. Adding a sentence or two to smoothly transition into the methods section could improve the overall flow of the introduction.

 Response: We added the following sentence to Introduction.

 “An average treatment effect model including propensity matching was applied to create these situations.”

 15. While the introduction mentions propensity score matching, a brief explanation of how it works and its purpose in addressing selection bias could enhance reader understanding.

 Response: We added the following sentence to Abstract.

 “We applied the average treatment effect model, particularly propensity scoring, which can treat the choice of administered drug as if administration were randomly assigned to inpatients.”

 16. The introduction could touch upon the external validity of the study, acknowledging that findings may not be universally applicable due to variations in healthcare systems, populations, or treatment protocols.

 Response: We added the following sentences to Introduction.

 ““Real-world effectiveness of drugs” means the effectiveness of drugs considering several factors including population characteristics, examinations, vaccinations, and healthcare system such as hospitalization and treatment protocols. Therefore, the results of this study might not be applicable to other countries.”

 17. The introduction could explicitly state the research question or hypothesis being addressed by the study to guide readers in understanding the study's purpose from the outset.

 Response: We added the following sentence to Introduction.

 “The objective of this study was evaluating the “real-world effectiveness of drugs against COVID-19 in Japan.””

 18. The text repeats certain points, such as the mention of "propensity score" and "saving life," multiple times. Reducing redundancy could improve the overall clarity and conciseness of the text.

 Response: We did the English proofreading.

 19. Some sentences are quite complex, which might make it challenging for readers to follow the arguments. Simplifying the language without compromising precision could enhance comprehension.

 Response: We did the English proofreading.

 20. The text occasionally uses language that implies causation, such as "might contribute to saving life." It's important to be cautious about making causal claims based on observational data. Acknowledging the limitations in establishing causation would strengthen the study's validity.

 Response: We added the following sentence to Discussion.

 “Third, this study was an observational study that was able to show only association between considered drugs and death, not necessarily a causal association.”

 21. The text mentions "not available" entries in the results for antibody cocktails, attributing it to a small sample size. Providing a more explicit explanation of why certain data is missing and its potential impact on the results would enhance transparency.

 Response: The approval of antibody cocktails was relatively late, and the number of prescriptions was simply low, so there is no potential impact. We added the following sentence in Discussion.

 “The antibody cocktail results included many “not available” entries, although drug effectiveness was confirmed in well-matched cases. The approval of antibody cocktails was late. The number of prescriptions was simply low, which might affect many cases for which “not available” was reported.”

 22. The limitations section is placed towards the end of the text. Moving it closer to the beginning or integrating limitations as they become relevant in the discussion could improve the flow and help readers contextualize the findings.

 Response: We integrated limitations to Discussion and changed each limitation to understand easily for the reader.

 23. The use of terms like "severely ill," "moderate severity," and "less–severe illness" could benefit from clearer operational definitions or references to specific criteria used for classification.

 Response: We added the following sentence to Method.

 “For this study, mild symptoms were defined as a patient who did not require medical intervention such as oxygen therapy or respiratory ventilation, moderate symptoms were defined as a patient who required oxygen administration, and severe symptoms were defined as a patient who required respiratory ventilation.”

 24. The discussion section covers a range of topics, including limitations and future research, without clear organization. Structuring the discussion into subsections could make it more reader-friendly.

 Response: We have organized discussion structure by issue.
